# Integrated Biological Responses Define the Thermoneutral Zone of Murrah Buffalo Calves in a Subtropical Climate

**DOI:** 10.3390/ani16182895

**Published:** 2026-09-14

**Authors:** Reetu Kumari, Priyambada Kumari, Brijesh Yadav, Shanker Kumar Singh, Vansh Sharma, Manish Tiwari, Arun Kumar Madan

**Affiliations:** 1 College of Biotechnology, Veterinary University (DUVASU), Mathura 281001, Uttar Pradesh, India; kreetu32@gmail.com (R.K.); priyambiochem@gmail.com (P.K.); vansh.8676@gmail.com (V.S.); bt.manish1@gmail.com (M.T.); 2 Department of Veterinary Physiology, Veterinary University (DUVASU), Mathura 281001, Uttar Pradesh, India; 3 Department of Veterinary Medicine, Veterinary University (DUVASU), Mathura 281001, Uttar Pradesh, India; pshankervet@gmail.com; 4 ICAR-Central Institute of Research on Buffalo, Hisar 125001, Haryana, India

**Keywords:** cold stress, critical temperature, heat stress, thermoneutral zone, threshold THI

## Abstract

The study was conducted to establish the thermoneutral zone (TNZ) of Murrah buffalo calves under precisely regulated environmental conditions. Six healthy calves (8–10 months) were exposed to progressively decreasing (24–15 °C; Temperature-Humidity Index (THI): 72.45–58.98) and increasing (25–40 °C; THI: 73.49–90.32) ambient temperatures with 3 °C intervals in a psychrometric chamber. Each exposure was maintained for 10 consecutive days under cyclic conditions (12 h exposure and 12 h thermoneutral recovery). Physiological responses and body surface temperature (BST) were recorded, and blood samples were collected on day 10 at 1500 h. Segmented regression analysis was performed using the SegReg (Oosterbaan, 2017) software program to identify lower and upper critical temperatures (LCT, UCT) and corresponding THI thresholds. LCTs ranged from 18.06 to 19.14 °C across different responsive parameters, with breakpoints (BPs) at 18.60 °C (SE_BP = 0.634) for pulse rate (PR), 18.15 °C (SE_BP = 0.945) for haemoglobin, 18.15 °C (SE_BP = 0.189) for lymphocytes, 18.24 °C (SE_BP = 0.404) for granulocytes, 19.14 °C (SE_BP = 1.09) for reactive oxygen species (ROS), 18.15 °C (SE_BP = 23.9) for superoxide dismutase (SOD), and 18.06 °C (SE_BP = 4.2) for cortisol; no distinct LCT was detected for respiratory rate (RR), rectal temperature (RT), total leukocyte count (TLC), or heat shock protein (HSP) 70/HSP90 expression. BST at different locations exhibited an LCT between ~20 and 21 °C. UCTs for PR, RR and RT were observed at 28.9 °C (SE_BP = 0.892), 28.6 °C (SE_BP = 0.843) and 29.2 °C (SE_BP = 1.16), respectively. Neither BST nor erythrocytic parameters exhibited a distinct UCT; however, BST increased progressively with increasing exposure temperature. TLC, lymphocyte and granulocyte % showed UCTs of 28.15 °C (SE_BP = 0.753), 28.15 °C (SE_BP = 0.497) and 32.65 °C (SE_BP = 14.20), respectively. The UCT for cortisol was observed at 28.15 °C (SE_BP = 5.52), whereas ROS and SOD showed UCTs of 28.75 °C (SE_BP = 4.67) and 34.15 °C (SE_BP = 6.55), respectively. HSP70 and HSP90 exhibited breakpoints at 35.35 °C (SE_BP = 1.32) and 34.30 °C (SE_BP = 2.11), respectively. Integration of key physiological and systemic responses (excluding BST) indicated a TNZ ranging from 19.14 °C to 28.15 °C. These findings provide a multi-level characterization of thermophysiological responses in buffalo calves and support the use of integrated biological indicators for defining thermoneutral conditions under controlled environments. The estimated TNZ of 19.14–28.15 °C provides a practical reference for managing Murrah buffalo calves. Producers should monitor housing temperature and consider ventilation, shade and air movement when temperatures approach or exceed ~28 °C while providing protection from cold when temperatures fall below ~19 °C. This may help reduce thermal strain and maintain normal physiological function.

## Simple Summary

This study comprehensively characterizes the thermoneutral zone of Murrah buffalo calves using integrated physiological, hematological, oxidative, endocrine, molecular, and infrared thermographic indicators under controlled environmental conditions. The thermoneutral zone was estimated at 19.14–28.15 °C, with biological responses indicating the onset of cold stress below 19.14 °C and heat stress above 28.15 °C (approximately corresponding to a Temperature–Humidity Index of 78). Body surface temperature was the earliest indicator of cold-related thermal responses, whereas physiological, oxidative, endocrine, and molecular responses showed distinct temperature thresholds during heat exposure. These findings provide practical thermal reference points for improving environmental management, animal welfare, and productivity in Murrah buffalo calves.

## 1. Introduction

The thermoneutral zone (TNZ) is defined as the range of ambient temperatures within which animals maintain physiological homeostasis without the need for additional energy expenditure for heat production or dissipation [1,2,3]. This zone is delimited by the lower critical temperature (LCT) and the upper critical temperature (UCT), beyond which animals initiate thermoregulatory responses to maintain core body temperature [4]. Exposure to temperatures below the LCT results in cold stress, while those above the UCT lead to heat stress, both of which compromise metabolic efficiency and overall health [5,6].

The TNZ has been defined for various livestock species using multiple physiological parameters and exhibits wide variation depending on the species, breed, age, and physiological status, with reported ranges of −1 to +10 °C for European cattle and 10 to 26 °C for Indian cattle [7] and 18 to 30 °C for zebu calves [8]. This variability between *Bos taurus* and *Bos indicus* cattle is primarily attributed to a lower LCT in *Bos taurus* and a higher UCT in *Bos indicus* [7]. Although *Bubalus bubalis* shares certain physiological similarities with *Bos indicus*, it differs from cattle in thermoregulatory efficiency due to distinct anatomical and physiological characteristics. The dark skin pigmentation of buffalo enhances absorption of solar radiation, while the comparatively lower density and activity of sweat glands restrict evaporative heat dissipation [9]. In addition, thicker skin and sparse hair coat reduce cutaneous heat loss efficiency, making buffaloes more dependent on behavioral cooling mechanisms such as wallowing [9,10,11]. These characteristics contribute to the higher susceptibility of buffaloes to both heat and cold stress and justify species-specific determination of the TNZ. Consequently, the threshold values of the temperature-humidity index (THI) used to define environmental stress also differ between buffaloes and zebu cattle. In cattle, THI values below 72 are considered thermoneutral, values of 72–78 indicate moderate stress, and values above 79 denote severe stress [12]. However, in buffaloes, the threshold THI has been reported to be above 80 [13].

Before the year 2000, TNZ was identified based on respiration rate (RR), pulse rate (PR), rectal temperature (RT) and production parameters [7,14]. In recent years, UCTs and threshold THIs have been established using biochemical and endocrine indicators [15,16,17,18], vaginal, ruminal, milk, and body surface temperatures [19,20,21,22], in addition to production parameters [17]. Although TNZ has traditionally been estimated using physiological indicators such as RT, RR and PR, these parameters may not fully capture thermal responses occurring at different levels of biological organisation. In our previous study, the TNZ and threshold THIs in Zebu calves were identified using a comprehensive assessment of physiological, hematological, biochemical, hormonal, and molecular stress parameters [8]. This integrated approach provides a broader assessment of thermal adaptation and may improve the characterization of critical temperature thresholds compared with reliance on conventional physiological indicators alone. While identifying the TNZ and critical THIs is essential, understanding the sequence of biological responses beyond these thresholds is equally important. We hypothesized that increasing thermal load would elicit differential responses across biological systems, with individual indicators exhibiting distinct temperature thresholds as environmental conditions deviated from the TNZ. Accordingly, the present study evaluated these responses to characterize the TNZ and associated thermal thresholds in Murrah buffalo calves.

Calves are particularly susceptible to cold stress because of their greater surface area relative to body mass and underdeveloped ruminal fermentation, which limits endogenous heat production [23,24]. Although brown adipose tissue contributes to heat production through non-shivering thermogenesis during early life, its quantity and functional efficiency decline rapidly with age. Consequently, neonatal and growing calves rely more on metabolic energy reserves and behavioral adaptations to maintain body temperature, increasing their susceptibility to cold environments outside the TNZ. Although heat stress thresholds are relatively higher in calves, once thermal stress is initiated, physiological responses tend to be stronger but less efficient than adult cattle [8]. However, studies systematically defining LCT, UCT, and corresponding THI thresholds using integrated physiological and biochemical indicators in buffaloes, particularly buffalo calves, remain limited.

The present study therefore moves beyond the simple detection of thermal stress and aims to identify the relative temperature thresholds and response patterns of physiological and systemic indicators as environmental conditions deviate from the TNZ, thereby characterizing the order in which different regulatory systems are challenged during thermal strain. Accordingly, the study was undertaken to determine the TNZ, threshold THIs, and the relative temperature thresholds and response patterns of physiological and systemic indicators in buffalo calves exposed to increasing and decreasing ambient temperatures, thereby contributing to the development of effective thermal stress mitigation strategies.

## 2. Materials and Methods

### 2.1. Place of Study

The current research was conducted at the Prof. M. D. Pandey Bio-Climatology Laboratory, Department of Veterinary Physiology, College of Biotechnology, DUVASU, Mathura, Uttar Pradesh, India. The study site is located at 78° E longitude and 27° N latitude, with an elevation of 176 m above sea level, within the semi-arid climatic zone of India. The region experiences annual minimum and maximum temperatures ranging from 4 °C to 46 °C, while mean annual relative humidity ranges between 25% and 85%. Annual precipitation varies from 200 to 400 mm and is characterized by an irregular seasonal distribution.

### 2.2. Animals

The experimental protocol was conducted in accordance with the guidelines of the Committee for Control and Supervision of Experiments on Animals (CCSEA) of the Government of India, following approval from the Institutional Animal Ethics Committee (IAEC) of the Veterinary University, Mathura (Approval No. IAEC/24/1/46, dated 5 March 2024). The study was conducted using six apparently healthy male Murrah buffalo calves, aged between 8 and 10 months and weighing between 120 and 150 kg, which were raised at the university’s Livestock Farm Complex. The body condition score (BCS) of the calves was recorded prior to the experiment and averaged 3.5 ± 0.3 (on a 5-point scale). Maintaining uniform body condition minimized variation in thermal insulation and its potential influence on LCT. The sample size was comparable to that used in previous controlled thermal stress studies in cattle and buffaloes evaluating physiological and molecular responses under climatic chamber conditions [8,10,16]. Furthermore, the repeated-measures design allowed each calf to serve as its own control across multiple environmental conditions, thereby increasing statistical efficiency and reducing the number of animals required. The buffalo calves were acclimatized for 10 days before the beginning of the actual experiment in a well-ventilated antechamber designed to accommodate 10 animals, which was equipped with individual feeding mangers, sprinklers, and fans to ensure proper airflow and cooling. The animals had free access to drinking water and were fed wheat straw supplemented with the requisite quantity of concentrate mixture as per the routine farm feeding practices. The same dietary regimen was maintained throughout the experimental period. Before initiation of the experiment, all animals were dewormed and subsequently immunized against Foot-and-Mouth Disease (FMD) and Haemorrhagic Septicemia as preventive health measures.

### 2.3. Experimental Design

The experiment was performed in two distinct phases using the psychrometric chamber: a cyclic heat-stress (HS) phase and a cyclic cold-stress (CS) phase. Before the experiment began in the psychrometric chamber, the calves were acclimatized to the chamber conditions for 10 days at 25 °C with 65% humidity. In the first phase of the experiment, six healthy calves were exposed to cyclic heat stress conditions inside a psychrometric chamber, where exposure temperatures were progressively increased from 25 to 40 °C in 3 °C increments (25, 28, 31, 34, 37, and 40 °C), corresponding to THI values ranging from 73 to 90. The calves were exposed to each temperature for 12 h daily (0600–1800 h), and during the remaining 12 h (1800–0600 h), they were maintained at 25 °C and 65% relative humidity (RH) (THI ≈ 73.5) within the same chamber. Each temperature–humidity combination was maintained for ten consecutive days to ensure steady-state adaptation and consistent data collection. Following completion of the heat stress phase, the animals were kept under normal environmental conditions for a recovery period of one month, during which no experimental stress was imposed. In the second phase, the same calves were subsequently exposed to cyclic cold stress conditions with progressively decreasing exposure temperature of 24, 21, 18 and 15 °C. The cold exposure trial was terminated at 15 °C when calves exhibited signs indicative of hypothermia, including persistent shivering, reduced activity, and a decline in rectal temperature of approximately 1.5 °C from baseline values. These criteria were applied to ensure animal welfare and to prevent progression to severe cold stress. Similar to the heat stress phase, calves were exposed to each cold temperature for 12 h daily (0600–1800 h), followed by a 12-h recovery period (1800–0600 h) at 25 °C and 65% RH. Each temperature was maintained for ten days, with corresponding THI values ranging between 72.5 and 59.0. Prior to the initial temperature exposure, the calves underwent a 10-day acclimation period at 25 °C in the chamber to stabilize their physiological responses. No recovery period was provided between successive temperature exposures, as the calves were exposed sequentially to simulate natural temperature progression and characterize their continuous biological responses. The temperature and relative humidity during distinct phases in the psychrometric chamber is presented in Table 1.

On the 10th day of every temperature exposure, physiological parameters and body surface temperature (BST) were recorded, and blood samples were collected at 1500 h. Desired combinations of temperature, relative humidity, and carbon dioxide were set every morning at 0600 h and 1800 h via the automation panel (Model No. PNL-JSP-SZ-90523-CON-1153; Controls Instruments, New Delhi, India). To achieve the desired environmental conditions while avoiding sudden changes in temperature, a temperature gradient of 2 °C at 15-min intervals was programmed so that the desired temperature and relative humidity combination was ultimately achieved. THI served as an indicator of environmental heat stress and was calculated using the formula proposed by National Research Council (1971) [25].

THI = (1.8 × Tdb + 32) − [(0.55 − 0.0055 × RH) × (1.8 × Tdb − 26.8)]

where Tdb is the dry-bulb temperature, and RH is the relative humidity.

### 2.4. Climatic Chamber

**Figure 1:**
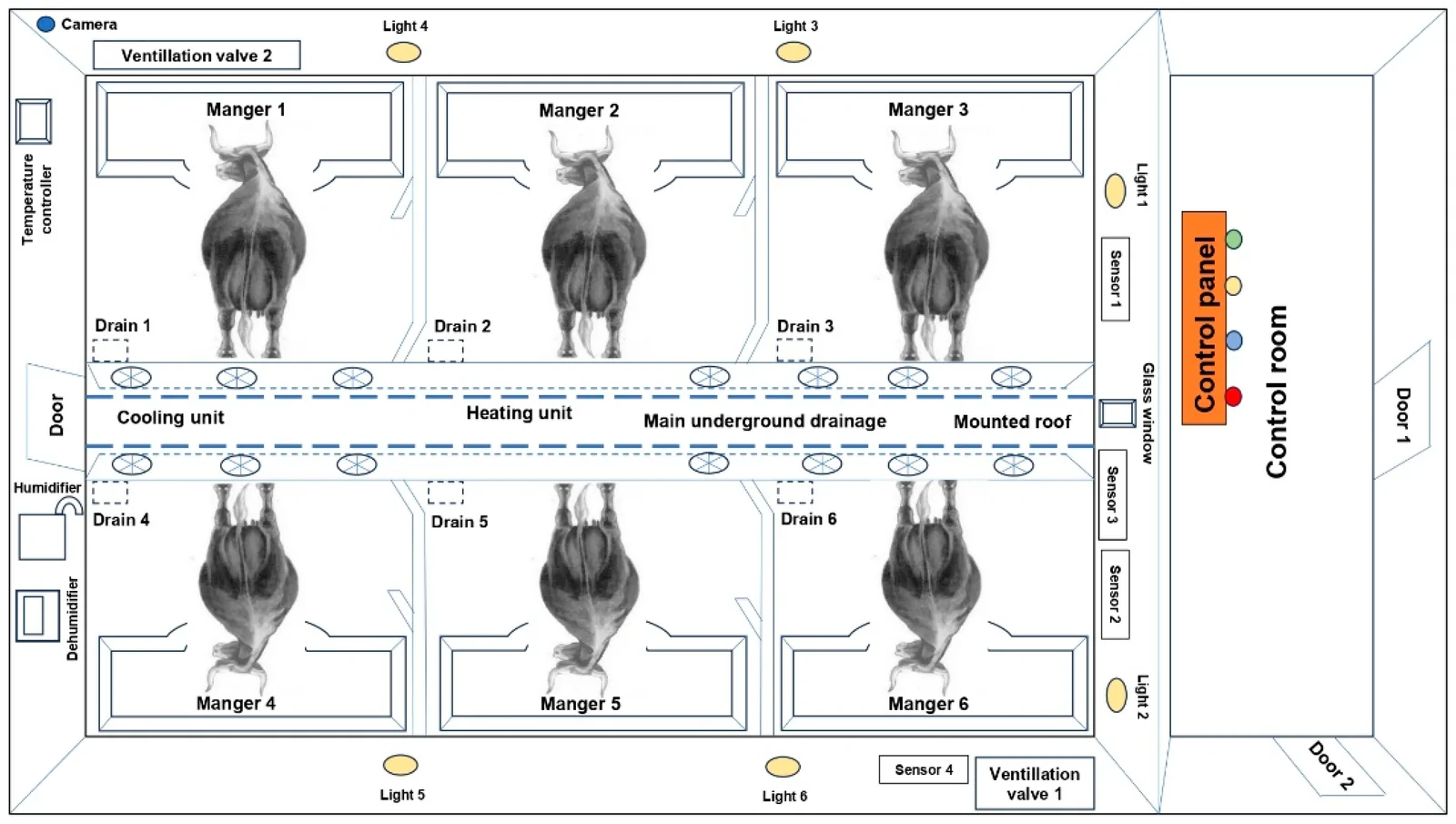
Schematic representation of the psychrometric chamber used for controlled temperature exposure of buffalo calves.

The climatic chamber is capable of maintaining controlled environmental conditions, with temperature adjustable from 5 °C to 55 °C and relative humidity from 15% to 75%, with an accuracy of ±1.0. The chamber measured 9.0 m × 4.0 m × 3.0 m and is equipped with individual tie-stalls and feeders to accommodate six adult animals. It was constructed using a steel framework with puffed insulation material to provide air tightness and effective thermal insulation. Ventilation is facilitated through diagonally positioned inlet and outlet valves, each fitted with exhaust fans. Sensors for temperature, relative humidity, carbon dioxide concentration, and light intensity are integrated with an external automation panel. The desired combinations of temperature, relative humidity, and carbon dioxide concentration are programmed through this panel, with approximately 10–30 min required to attain the specified environmental conditions. The chamber is operated for at least 30 min before initiating each designated temperature exposure. Environmental variables, including temperature, humidity, and carbon dioxide concentration, are monitored at 5-min intervals and recorded automatically through a data logger. The schematic representation of the psychrometric chamber is presented in Figure 1.

### 2.5. Physiological Observations

Pulse rate (PR) was determined by palpating the middle coccygeal artery at the tail base and expressed as beats per minute. Respiration rate (RR) was assessed by counting flank movements and reported as breaths per minute. Rectal temperature (RT) was measured in degrees Celsius (°C) using a K-Life KLF-102 digital clinical thermometer (K-Life Healthcare Pvt. Ltd., Gurugram, India), which was inserted rectally for 1–2 min.

### 2.6. Infrared Thermography

The BST of buffalo calves was recorded using infrared thermography (IRT). Thermal images were captured with FLIR infrared camera (A320 Series; FLIR Systems Inc., Wilsonville, OR, USA), with each recording session lasting approximately 3–5 min per animal. The camera featured a resolution of 464 × 348 pixels, thermal sensitivity of 0.03 °C, measurement accuracy of ±2% of the recorded value, a temperature detection range of −20 °C to 1800 °C, and a field of view of 24° × 18°. Imaging was carried out from a distance of 1.0–1.5 m while calves were restrained in a crate to minimize movement [26]. Six anatomical regions—muzzle, forehead, eyeball, ears, neck, and croup—were consistently targeted. Before imaging, ambient temperature and relative humidity were entered into the camera system. The reflected apparent temperature was maintained at 30 °C, while an emissivity value of 0.98 was used, corresponding to mammalian skin [27]. Prior to image acquisition, the infrared camera was acclimatized to the environmental chamber temperature to ensure measurement accuracy. During thermographic recording, artificial lights were switched off to minimize infrared reflection and thermal interference. For image analysis, a circular region of interest (ROI) was delineated around the eyeball, while rectangular ROIs were selected for the remaining body sites. Infrared temperature values were extracted from each ROI, and all thermograms were analyzed using FLIR Tools software (version 6.4). The results were expressed in degrees Celsius (°C).

### 2.7. Blood Sampling

Blood samples (5–6 mL) were obtained from the jugular vein through venipuncture while minimizing handling-related stress. The samples were collected in EDTA-coated vacutainer tubes (BD, Franklin Lakes, NJ, USA) for plasma separation, with 1 mL of each sample allocated for hematological analysis. The remaining blood fraction was utilized for the isolation of peripheral blood mononuclear cells (PBMCs).

### 2.8. Hematological Analysis

One millilitre of blood from each sample was analyzed using an automated hematology analyzer (Nihon Kohden, Tokyo, Japan; Model MEK-6550K). The assessed parameters included total leukocyte count (×10^3^/µL), red blood cell count (×10^6^/µL), haemoglobin (g/dL), packed cell volume (%), lymphocyte %, and granulocyte %.

### 2.9. Redox Status and Hormonal Status

Bovine-specific ELISA kits (Cat. No. E0110Bo; Bioassay Technology Laboratory, Shanghai, China) were employed to quantify plasma cortisol concentrations following the manufacturer’s instructions. Plasma reactive oxygen species (ROS) levels were evaluated based on hydrogen peroxide concentration using the 96-well plate assay described by Brambilla et al. (2001) [28]. Superoxide dismutase (SOD) activity in plasma was determined using a bovine-specific ELISA kit (Cat. No. E0003Bo; Bioassay Technology Laboratory, Shanghai, China).

### 2.10. Expression Analysis of HSP70 and HSP90 Genes from PBMCs

Peripheral blood mononuclear cells (PBMCs) were obtained from freshly collected blood by density-gradient separation using Histopaque-1077 (Sigma-Aldrich, Burlington, MA, USA) under aseptic conditions. The recovered cells were washed with PBS (pH 7.4), and the resulting pellet was subjected to total RNA extraction using the TRIzol-based procedure described previously [29]. RNA concentration and purity were determined spectrophotometrically, with an A260/A280 ratio exceeding 1.9, while RNA integrity was verified by agarose gel electrophoresis based on the presence of distinct 28S and 18S rRNA bands. First-strand cDNA was generated from 250 ng total RNA using the RevertAid First Strand cDNA Synthesis Kit (Thermo Fisher Scientific, Waltham, MA, USA) following the manufacturer’s protocol. PCR conditions were optimized for the *HSP70*, *HSP90*, and GAPDH genes using DreamTaq PCR Master Mix, with annealing temperatures of 60 °C for the *HSP* genes and 63.3 °C for GAPDH. Primer sequences, amplicon sizes, and gene accession numbers are provided in Table 2. PCR products were examined by electrophoresis on a 1% agarose gel. Relative mRNA expression of *HSP70* and *HSP90* was subsequently quantified by real-time quantitative PCR using PowerUp™ SYBR™ Green Master Mix (Bio-Rad, Hercules, CA, USA), with GAPDH serving as the reference gene. Each reaction was performed in triplicate, and relative transcript abundance was calculated using the 2^−ΔΔCt^ method described by Livak [30].

### 2.11. Statistical Analysis

Segmented regression analysis was performed using the SegReg program (Oosterbaan, 2017) [31] to characterize the relationships between exposure temperature or THI and the measured physiological, thermographic, hematological, oxidative, endocrine and molecular response variables and to identify the corresponding temperature or THI breakpoints. SegReg evaluates alternative regression function types (Types 1–6), representing different combinations of linear and horizontal segments, with or without a discontinuity at the breakpoint. Type 1 represents a single linear relationship without a breakpoint, whereas Types 2–6 represent alternative segmented relationships. The appropriate function type and corresponding breakpoint are determined by the statistical fit and significance criteria implemented in the program. Thus, a breakpoint was not imposed when the data did not support a segmented relationship.

For each response variable, exposure temperature was used as the independent variable to estimate the LCT during decreasing-temperature exposure and the UCT during increasing-temperature exposure. THI was analysed separately as the independent variable to obtain the corresponding lower and upper THI thresholds. The breakpoint (BP) was defined as the temperature or THI at which the fitted regression relationship changed between the segments. For each fitted relationship, the function type, number of observations (n), mean X, mean Y, slope (A), intercept (C), coefficient of explanation (CE), standard deviation of the regression coefficient, standard deviation of Y, standard deviation of residual Y, and standard deviation of X were obtained from the SegReg output. The statistical significance of the fitted regression relationship and breakpoint was evaluated using the analysis of variance (ANOVA) and significance testing implemented in SegReg, with *p* < 0.05 considered statistically significant. The uncertainty associated with the estimated breakpoint was also obtained from the SegReg output and reported using the available confidence limits. In the graphical outputs, individual symbols represent the observed data points, solid lines represent the fitted regression relationship or segmented regression segments, dashed lines surrounding the fitted relationship represent the 95% confidence belt where available, and the vertical dashed line indicates the estimated breakpoint (BP). For Type 1 relationships, no statistically supported breakpoint was identified.

For increasing-temperature exposure (25–40 °C), a statistically supported breakpoint in a response variable was interpreted as the UCT, whereas for decreasing-temperature exposure (24–15 °C), a statistically supported breakpoint was interpreted as the LCT. Where the segmented analysis did not provide a statistically supported breakpoint, the response was considered to not exhibit a distinct LCT or UCT within the experimental temperature range. The corresponding THI threshold was determined from the breakpoint and the temperature–humidity conditions recorded at that exposure.

### 2.12. Mathematical Output

The LCT and UCT values obtained for each parameter were plotted sequentially on a temperature scale to identify the highest LCT and lowest UCT among the assessed parameters. The temperature range between the highest LCT and lowest UCT was considered the TNZ. Parameters included in TNZ determination were selected based on biological relevance to core thermoregulation. BST was excluded from final TNZ estimation because it reflects peripheral heat exchange rather than internal homeostatic regulation.

## 3. Results

Segmented regression analysis using SegReg identified different temperature–response relationships among the physiological, thermographic, hematological, oxidative, endocrine and molecular variables. The analysis provided the corresponding function type, regression coefficients, coefficient of explanation (CE), breakpoint estimates, and standard error of the breakpoint (SE_BP). Complete SegReg outputs for decreasing and increasing-temperature exposures are presented in Supplementary Tables S1 and S2, respectively. For variables showing a statistically supported change in the temperature–response relationship, the estimated breakpoint was considered the corresponding LCT during decreasing-temperature exposure or UCT during increasing-temperature exposure.

**Figure 2:**
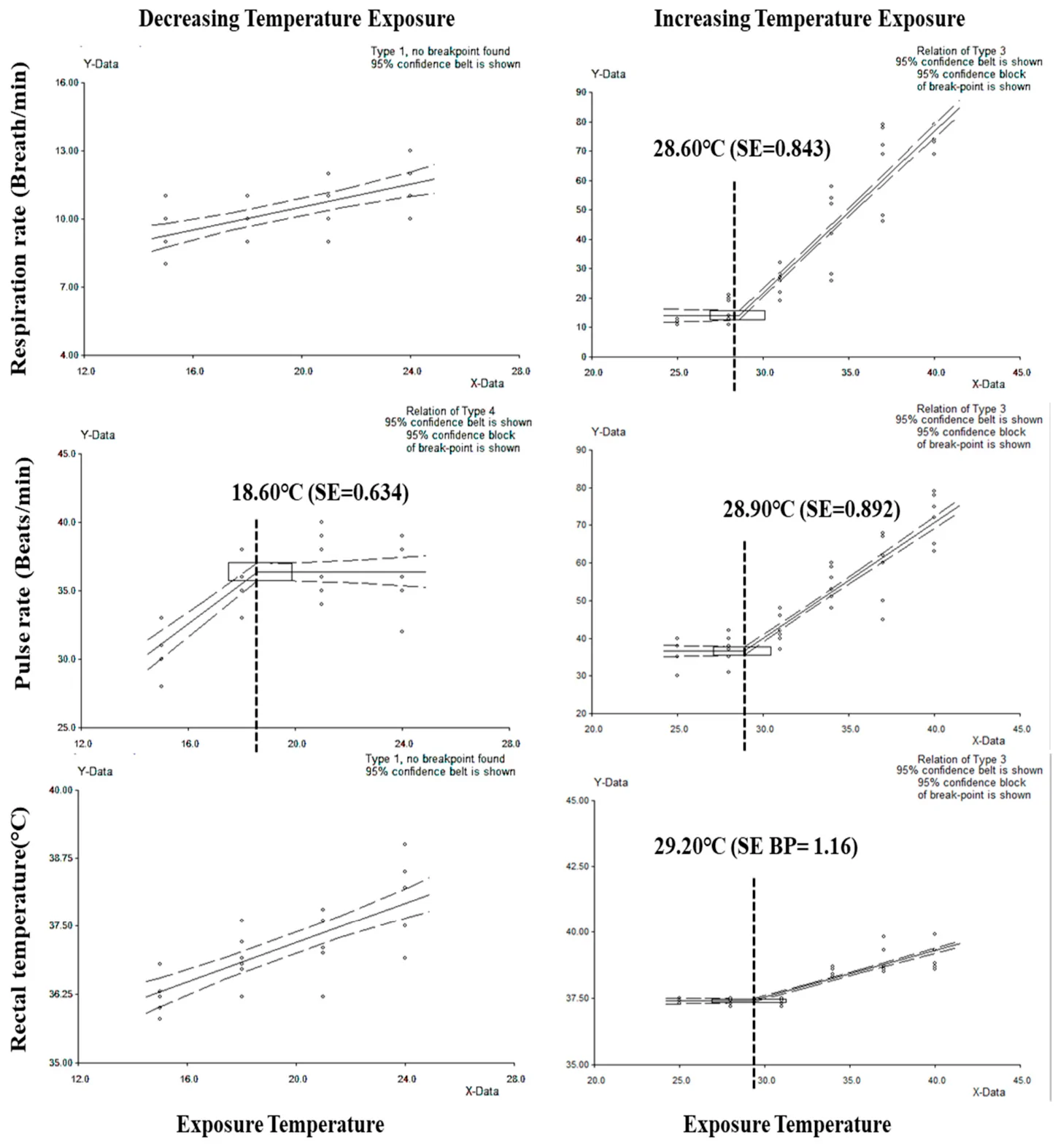
Best-fit segmented regression with breakpoints between different physiological parameters and exposure temperature. Individual points represent observed values, solid lines represent fitted regression relationships, dashed lines represent the 95% confidence belts, vertical dashed lines indicate the estimated breakpoints, and rectangular boxes indicate the 95% confidence blocks of the estimated breakpoints.

Segmented regression of physiological responses across the decreasing temperature range (24–15 °C) identified the corresponding temperature breakpoints as estimates of the LCT. The breakpoint (BP) for PR occurred at 18.60 °C (SE_BP = 0.634 °C), as illustrated in Figure 2, whereas no LCT was observed for RR and RT. The corresponding lower THI threshold for PR was 64.50 (SE_BP = 1.03) (Table 3). The RR and RT decreased progressively with decreasing exposure temperature (Figure 2). Likewise, the relationships between the physiological responses and increasing temperature (25–40 °C) and THI were examined using segmented regression to estimate the UCT and corresponding upper THI threshold. The UCT breakpoints for RR, PR, and RT were identified at 28.60 °C (SE_BP = 0.843 °C), 28.90 °C (SE_BP = 0.892 °C), and 29.20 °C (SE_BP = 1.16 °C), respectively (Figure 2). Correspondingly, the upper THI thresholds were 78.03 (SE_BP = 0.940) for RR, 78.37 (SE_BP = 1.09) for PR, and 78.71 (SE_BP = 1.35) for RT (Table 3).

**Figure 3:**
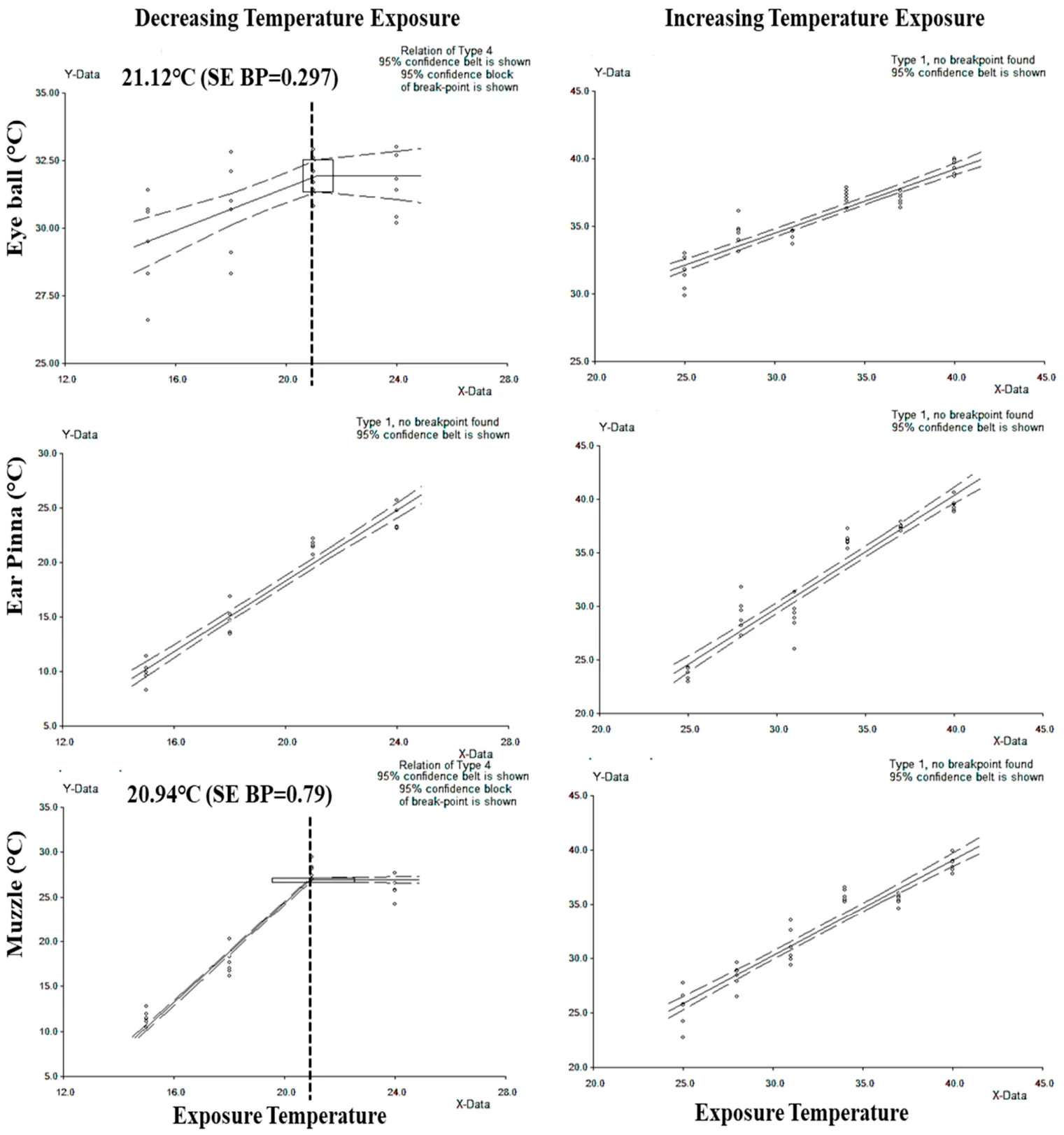

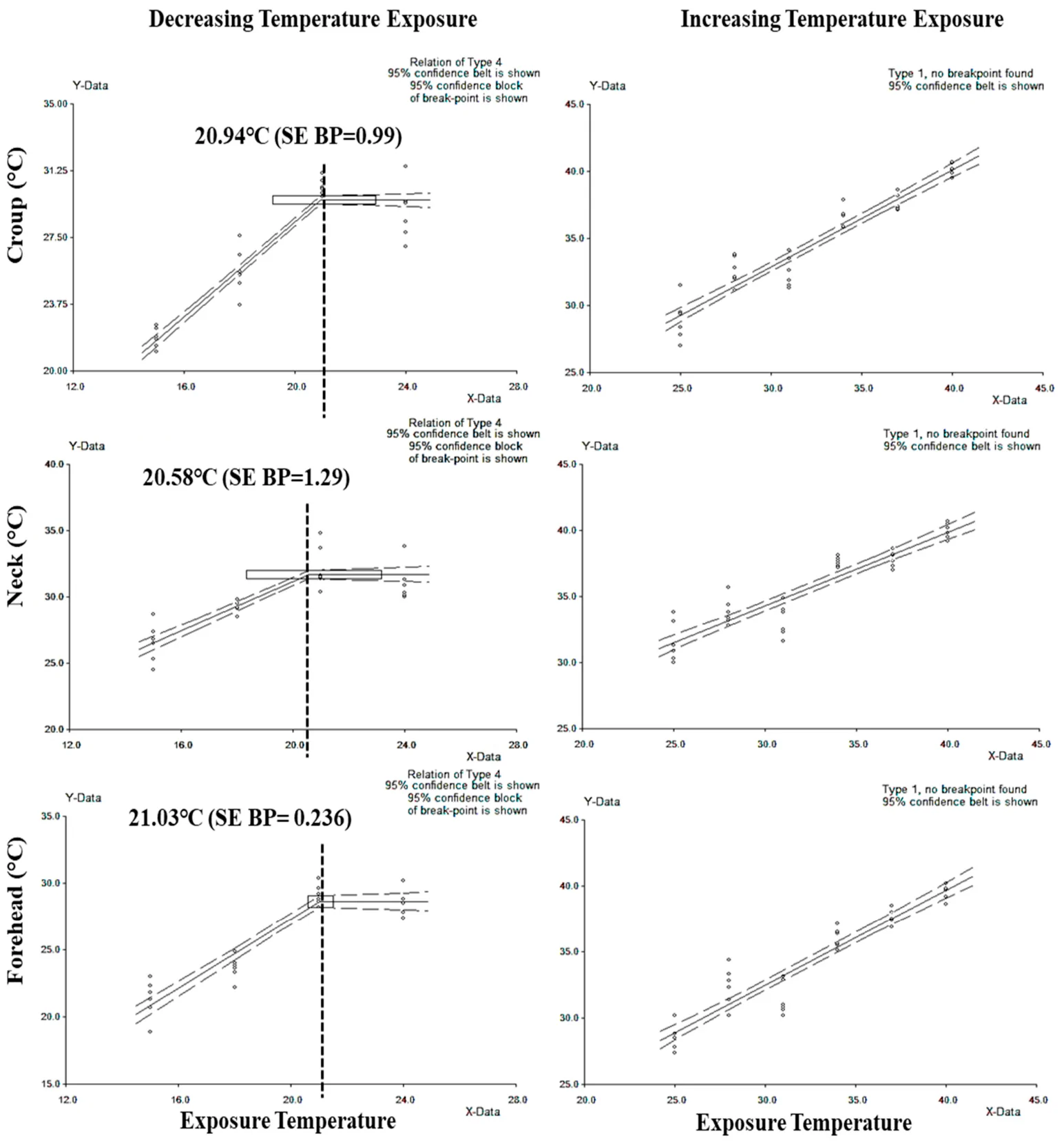
Best-fit segmented regression with breakpoints between different anatomical locations and exposure temperature. Individual points represent observed values, solid lines represent fitted regression relationships, dashed lines represent the 95% confidence belts, vertical dashed lines indicate the estimated breakpoints, and rectangular boxes indicate the 95% confidence blocks of the estimated breakpoints.

Figure 3 presents the temperature breakpoints corresponding to the LCT and UCT for the different BST parameters, whereas the associated lower and upper THI thresholds are presented in Table 3. The LCT values were identified as follows: 21.12 °C for the eyeball (SE_BP = 0.297 °C), 20.94 °C for the muzzle (SE_BP = 0.79 °C), 20.94 °C for the croup (SE_BP = 0.99 °C), 20.58 °C for the neck (SE_BP = 1.29 °C), and 21.03 °C for the forehead (SE_BP = 0.236 °C), whereas no significant (*p* > 0.05) breakpoint was identified for the ear pinna. The corresponding lower THI thresholds were 68.14 (SE_BP = 0.488) for the eyeball, 68.00 (SE_BP = 1.34) for the muzzle, 67.87 (SE_BP = 1.46) for the croup, 67.60 (SE_BP = 2.00) for the neck, and 68.00 (SE_BP = 0.446) for the forehead, with no threshold identified for the ear pinna. For the UCT, no distinct breakpoints were detected across the anatomical locations; BST increased progressively with increasing temperature.

**Figure 4:**
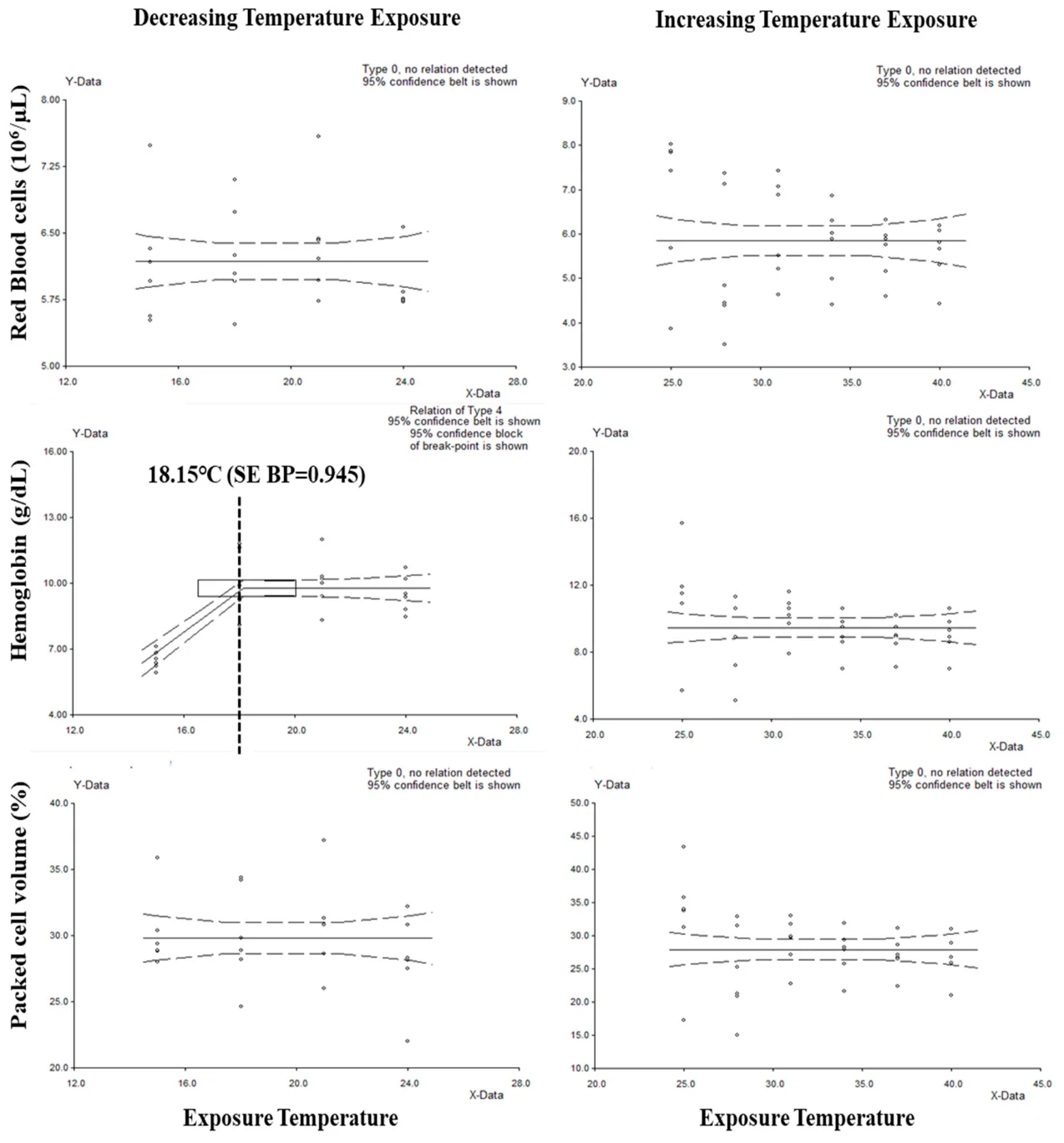
Best-fit segmented regression with breakpoints between different erythrocytic parameters and exposure temperature. Individual points represent observed values, solid lines represent fitted regression relationships, dashed lines represent the 95% confidence belts, vertical dashed lines indicate the estimated breakpoints, and rectangular boxes indicate the 95% confidence blocks of the estimated breakpoints.

Figure 4 illustrates the LCT and UCT breakpoints identified for the erythrocytic parameters, while the corresponding lower and upper THI thresholds are provided in Table 3. The LCT for haemoglobin was identified at 18.15 °C (SE_BP = 0.945 °C), whereas no breakpoints were observed for red blood cells (RBC) and packed cell volume (PCV). No UCT breakpoints were observed for erythrocytic parameters. Similarly, the lower THI threshold for haemoglobin was 63.83 (SE_BP = 1.45), while no significant upper THI thresholds (*p* > 0.05) were observed for any of the three erythrocytic parameters (Table 3).

**Figure 5:**
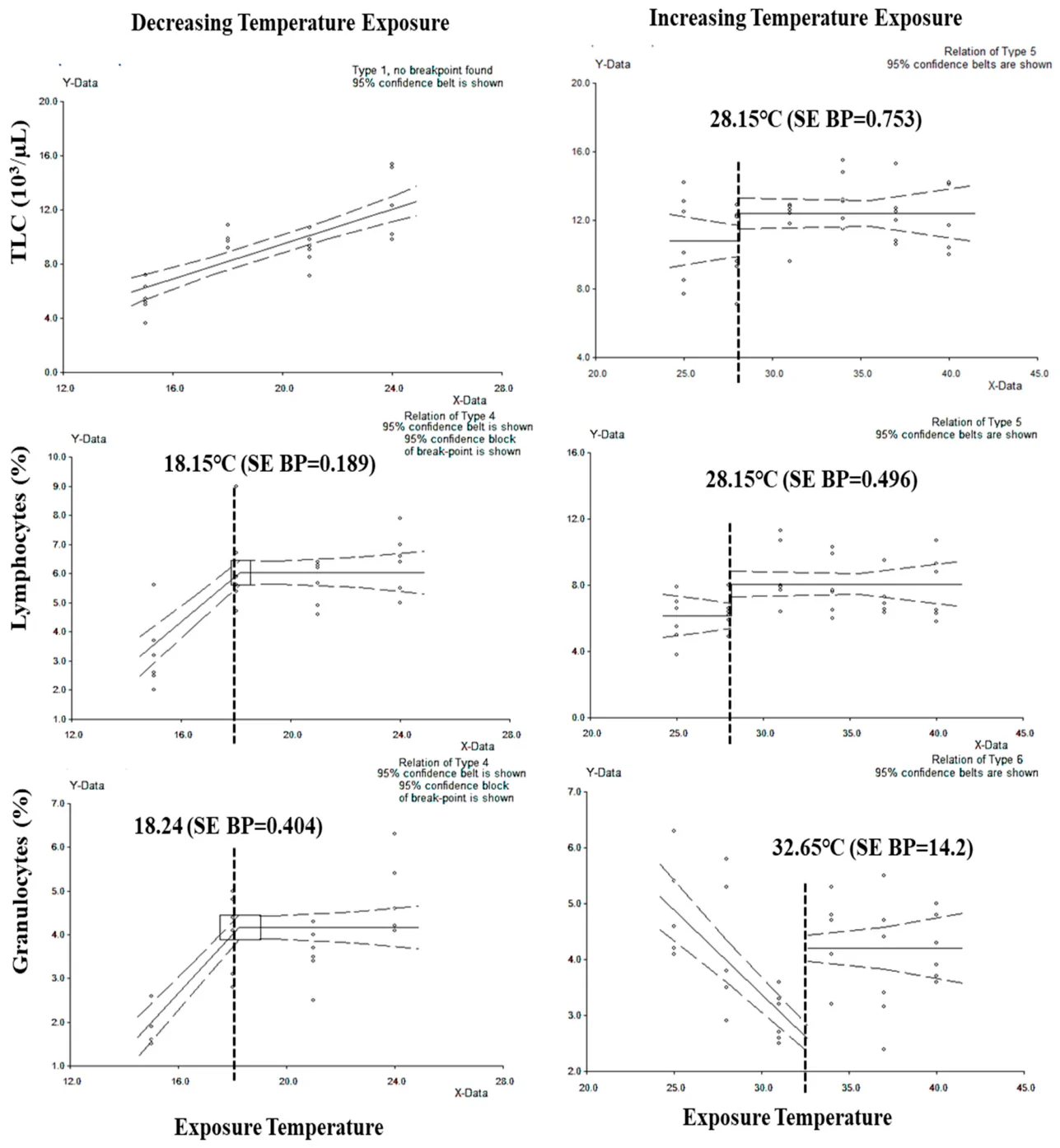
Best-fit segmented regression with breakpoints between different leukocytic parameters and exposure temperature. Individual points represent observed values, solid lines represent fitted regression relationships, dashed lines represent the 95% confidence belts, vertical dashed lines indicate the estimated breakpoints, and rectangular boxes indicate the 95% confidence blocks of the estimated breakpoints.

Figure 5 and Table 3 present the LCT and UCT breakpoints identified for the leukocytic parameters, together with their corresponding lower and upper THI thresholds, respectively. LCTs for lymphocyte and granulocyte percentage were observed at 18.15 °C (SE_BP = 0.189 °C) and 18.24 °C (SE_BP = 0.404 °C), respectively, whereas no LCT was identified for TLC. The lower THI thresholds for lymphocyte and granulocyte percentage were 63.96 (SE_BP = 0.313) and 63.83 (SE_BP = 0.651), while no significant lower THI threshold was detected for TLC (*p* > 0.05). The UCT for TLC was observed at 28.15 °C (SE_BP = 0.753 °C); however, lymphocyte and granulocyte percentage exhibited UCTs at 28.15 °C (SE_BP = 0.496 °C) and 32.65 °C (SE_BP = 14.2 °C), respectively. The corresponding upper THI thresholds for lymphocyte percentage, granulocyte percentage, and TLC were 77.36 (SE_BP = 0.496), 82.41 (SE_BP = 39.20), and 77.36 (SE_BP = 5.52), respectively.

**Figure 6:**
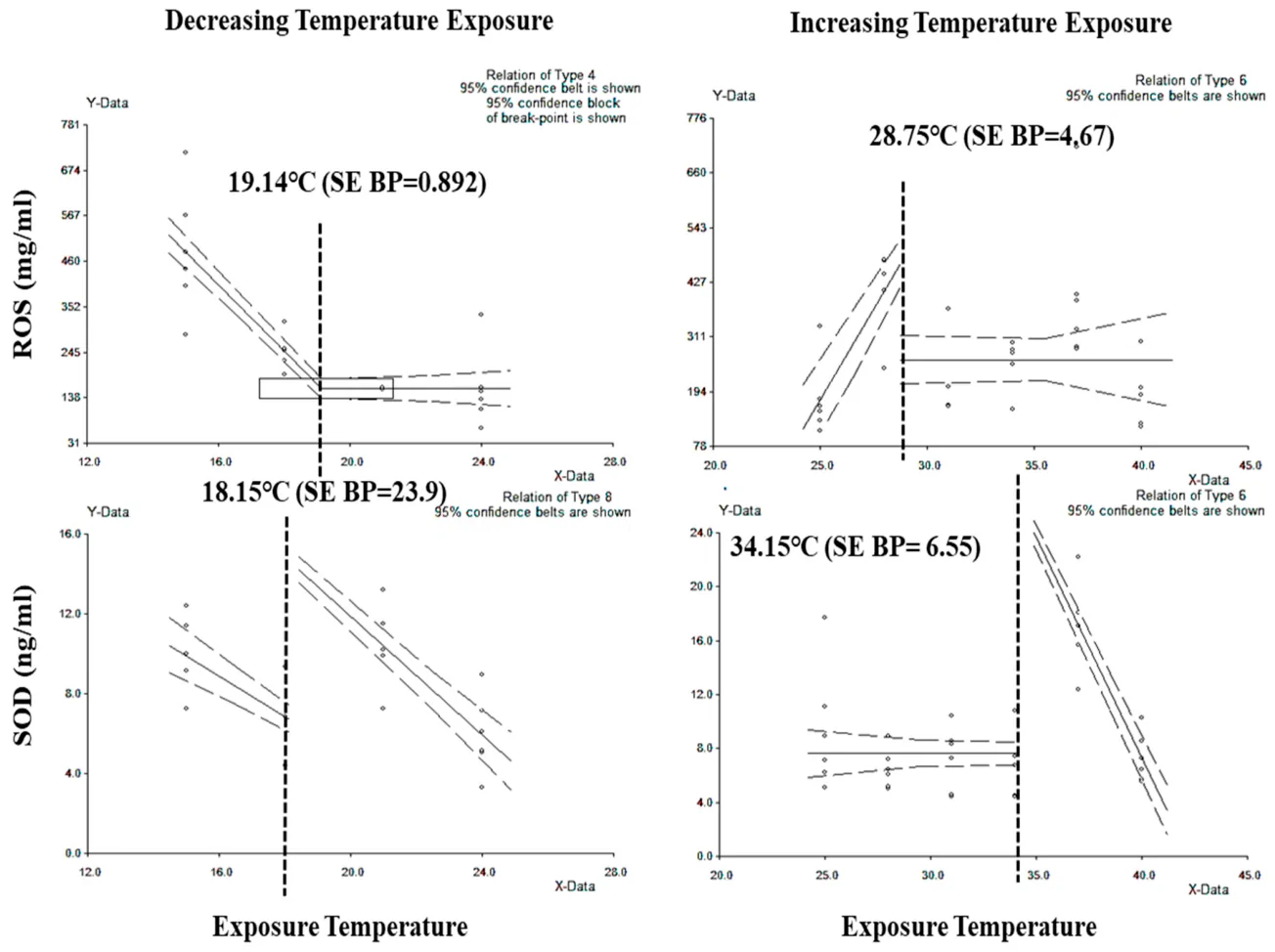
Best-fit segmented regression with breakpoints between different redox parameters and exposure temperature. Individual points represent observed values, solid lines represent fitted regression relationships, dashed lines represent the 95% confidence belts, vertical dashed lines indicate the estimated breakpoints, and rectangular boxes indicate the 95% confidence blocks of the estimated breakpoints.

The LCTs for ROS and SOD activity were identified at 19.14 °C (SE_BP = 1.09 °C) and 18.15 °C (SE_BP = 23.9 °C), respectively. Similarly, the UCTs for ROS and SOD were identified at 28.75 °C (SE_BP = 4.67 °C) and 34.15 °C (SE_BP = 6.55 °C), respectively (Figure 6). The corresponding lower THI thresholds for ROS and SOD were 65.31 (SE_BP = 1.09) and 63.83 (SE_BP = 23.9), while the upper THI thresholds were 78.03 (SE_BP = 19.50) for ROS and 83.76 (SE_BP = 15.0) for SOD activity (Table 3).

**Figure 7:**
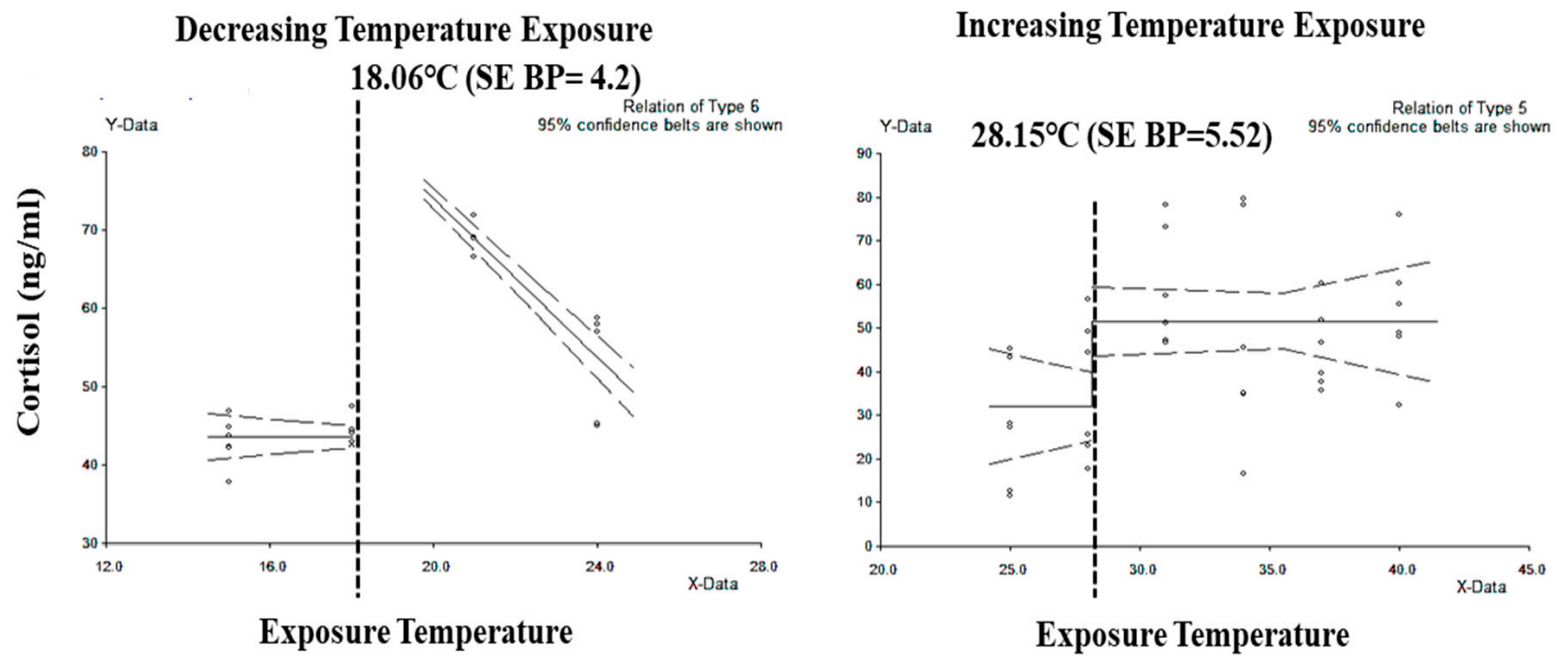
Best-fit segmented regression with breakpoints between cortisol and exposure temperature. Individual points represent observed values, solid lines represent fitted regression relationships, dashed lines represent the 95% confidence belts and vertical dashed lines indicate the estimated breakpoints.

The LCT for cortisol was observed at 18.06 °C (SE_BP = 4.2 °C), while its UCT was identified at 28.15 °C (SE_BP = 5.52 °C) (Figure 7). Correspondingly, the lower and upper THI thresholds for cortisol were 63.69 (SE_BP = 13.30) and 77.39 (SE_BP = 5.52), respectively (Table 3).

**Figure 8:**
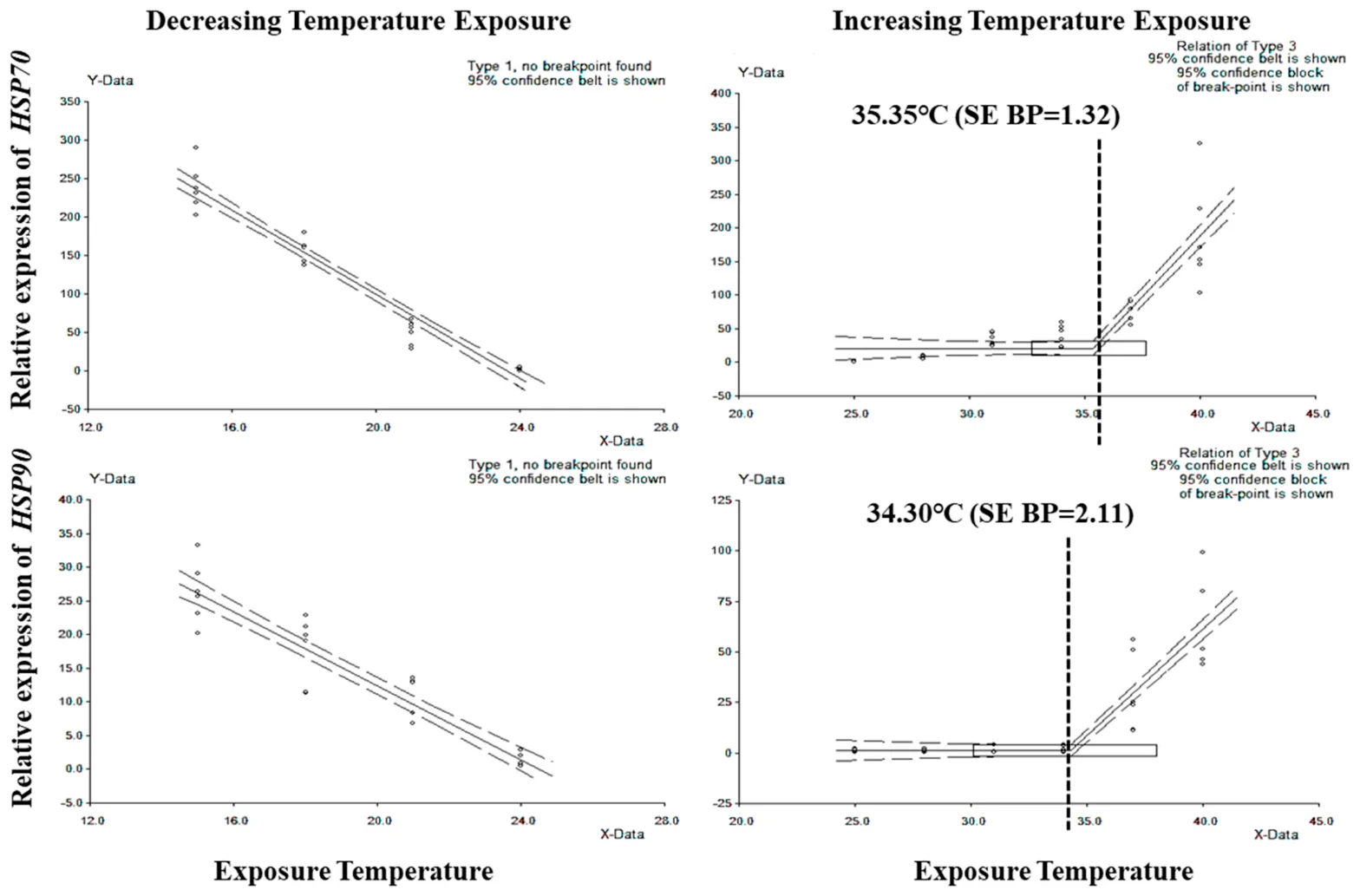
Best-fit segmented regression depicting the relationship between relative gene expression of *HSP*70 and *HSP*90 and exposure temperatures. Individual points represent observed values, solid lines represent fitted regression relationships, dashed lines represent the 95% confidence belts, vertical dashed lines indicate the estimated breakpoints, and rectangular boxes indicate the 95% confidence blocks of the estimated breakpoints.

Figure 8 and Table 3 show the relative gene expression patterns of *HSP*70 and *HSP*90. *HSP*70 and *HSP90* showed statistically significant (*p* < 0.05) variation, with a clear breakpoint identified at 35.35 (SE_BP = 1.32 °C) and 34.30 °C (SE_BP = 2.11 °C), respectively, while no LCT breakpoint was identified for either gene, although their expression decreased progressively with decreasing exposure temperature. The corresponding upper THI thresholds for *HSP*70 and *HSP90* were 85.44 (SE_BP = 1.35) and 84.33 (SE_BP = 2.17), whereas no lower THI breakpoint was detected for either gene (Table 3).

**Figure 9:**
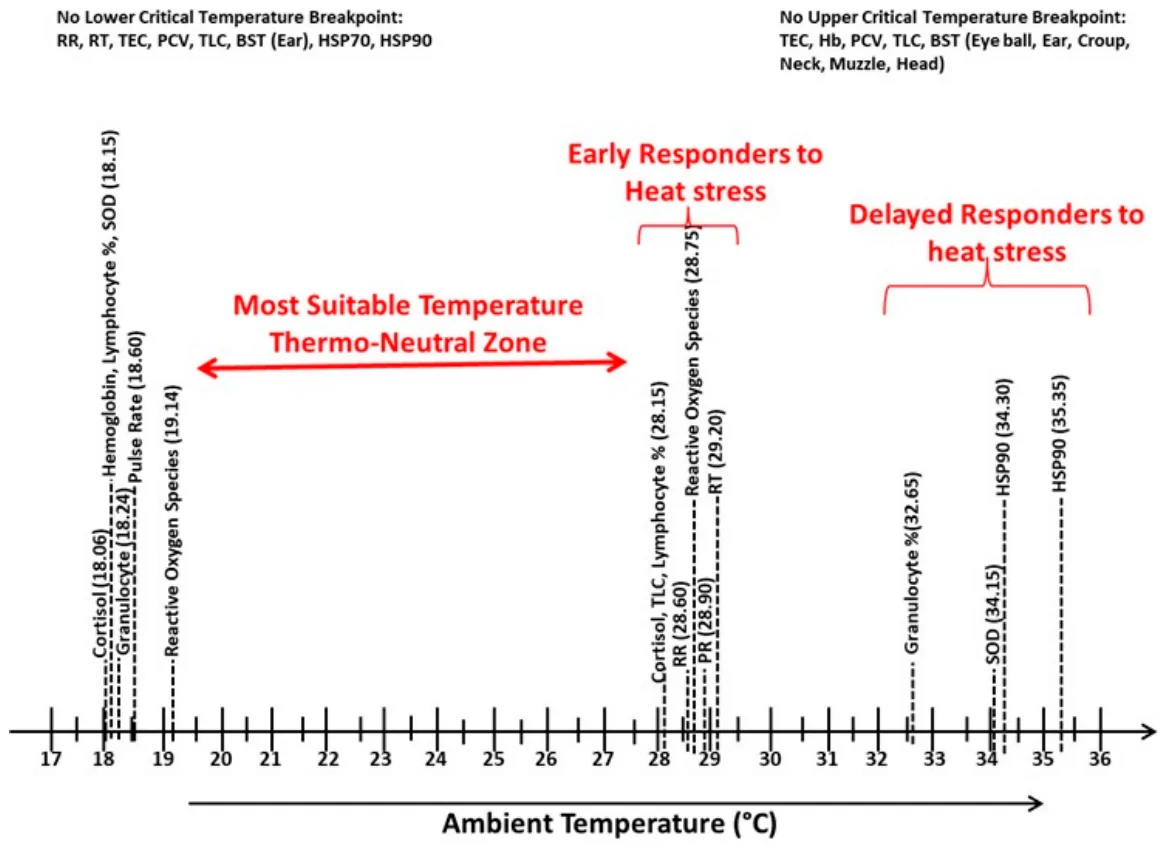
Operational thermoneutral zone (TNZ) estimated from the highest statistically supported lower critical temperature (LCT) and lowest statistically supported upper critical temperature (UCT) among the assessed biological indicators. The horizontal axis represents ambient temperature; vertical markers indicate individual LCT and UCT estimates. The red arrow denotes the estimated TNZ, while the “early” and “delayed responders” groups indicate the relative timing of statistically supported responses to increasing temperature. Parameters without statistically supported LCT or UCT are indicated separately.

As shown in Figure 9, parameters without statistically supported breakpoints either showed progressive temperature-dependent changes without a distinct change in slope or exhibited little or no appreciable change across the tested temperature range. To identify the temperature range associated with minimal changes across the assessed parameters, the LCT and UCT values were plotted sequentially on a common temperature scale (Figure 9). The operational TNZ was estimated as 19.14–28.15 °C based on the highest statistically supported LCT and lowest UCT among the assessed response variables. This approach follows the physiological concept of TNZ. No parameter was prioritized a priori based on its physiological, hematological, oxidative, endocrine, molecular or thermographic nature; rather, all assessed variables were considered equally, and those exhibiting statistically supported temperature breakpoints contributed to defining the respective TNZ boundary.

## 4. Discussion

In a previous study from our group, the TNZ in zebu calves was delineated using a comprehensive set of twenty physiological indicators [8]. However, these thresholds cannot be directly extrapolated to buffalo calves because of inherent anatomical and physiological differences between cattle and buffalo [9,10,11]. Thermal vulnerability is further accentuated during early life stages, as Murrah buffalo calves possess immature thermoregulatory mechanisms, sparse hair covering, and relatively low subcutaneous fat insulation, all of which compromise their ability to conserve metabolic heat under cold exposure [23,24]. Under Indian production conditions, exposure to low ambient temperatures during winter months remains a significant contributor to calf morbidity and mortality [32]. Consequently, establishing species-specific estimates of lower and upper critical temperatures and the corresponding TNZ is essential for accurately characterizing thermal tolerance in buffalo calves and for developing evidence-based management strategies aimed at minimizing thermal stress and improving calf survival and productivity.

The physiological responses observed in the present study indicated that PR was the earliest parameter to respond to declining ambient temperature. Below the LCT of 18.60 °C, PR declined progressively, suggesting reduced peripheral circulation and metabolic activity as part of the heat-conservation mechanisms during cold exposure [33,34]. In contrast, RR and RT showed no distinct breakpoints and decreased gradually with declining temperature, indicating progressive cold strain rather than stable thermoregulatory compensation. Similar responses have been reported in cattle and Sahiwal calves, where PR was the most sensitive physiological indicator of cold stress [8,34,35,36]. The slightly higher LCT observed in Murrah buffalo calves suggests greater susceptibility to cold stress, likely due to their lower capacity for endogenous heat production during early life. With increasing environmental temperature, RR exhibited the earliest breakpoint, followed by PR and RT, indicating differences in the temperature thresholds of these physiological responses as heat load increased. The onset of physiological heat strain occurred when THI approached 78. Increased RR enhances evaporative heat dissipation, while elevated PR and RT reflect increased circulatory adjustments and reduced the ability to maintain thermal balance [10,17,37]. THI induced RR, PR and RT responses in Murrah buffaloes that showed a phasic rather than strictly linear pattern, with marked physiological changes becoming evident at higher THI levels, particularly around THI 77–80 [38,39]. Overall, PR showed the earliest statistically supported response to declining temperature, whereas RR, PR and RT exhibited distinct temperature thresholds during heat exposure.

Infrared thermography revealed distinct lower critical thresholds for BST, indicating that peripheral tissues are highly sensitive to declining ambient temperature [26,40]. The observed reductions likely reflect peripheral vasoconstriction, which reduces cutaneous blood flow and minimizes heat loss [26]. Because peripheral tissues are directly exposed to the environment, BST responded earlier than core physiological variables, suggesting its utility as an indicator of peripheral thermoregulatory responses during cold exposure. The decline in BST with decreasing environmental temperature is consistent with the previous findings in Murrah buffaloes, which showed reduced peripheral surface temperature during extreme cold [40]. In contrast, BST increased linearly with rising ambient temperature and did not exhibit a distinct upper critical threshold, indicating that it primarily reflects environmental heat load rather than intrinsic thermoregulatory limits.

Hemogram and leukogram parameters are widely used indicators of thermal stress in livestock [41]. In the present study, cold exposure reduced haemoglobin concentration at 18.15 °C, whereas RBC count and packed cell volume remained unchanged, contrasting with the increases reported in cold-stressed buffalo calves [33]. Heat stress similarly had no significant effect on hemogram parameters, supporting earlier reports that erythrocytic indices remain relatively stable under moderate thermal stress [42]. Among leukogram variables, lymphocyte percentage was the most sensitive indicator, responding earliest to both cold and heat stress, followed by granulocyte percentage. These findings agree with previous reports demonstrating the thermal sensitivity of leukocyte profiles in cattle and buffaloes [18,24,43]. Under heat stress, the breakpoint in lymphocyte percentage at 28.15 °C occurred at a lower temperature than that observed for granulocyte percentage, indicating differential sensitivity of leukocyte populations to increasing thermal load [44,45]. Overall, the results identify lymphocyte percentage as the most responsive leukogram marker of thermal stress in buffalo calves.

ROS and SOD responses provide important indicators of oxidative stress and antioxidant defence under thermal challenge [15]. In the present study, the LCTs identified for ROS and SOD suggest that mild cold exposure was associated with an increase in ROS production, accompanied by SOD activation as part of the antioxidant defence response. During heat stress, the earlier breakpoint observed for ROS relative to SOD is consistent with a possible transient imbalance between ROS generation and antioxidant capacity, followed by a compensatory antioxidant response [11,17].

Heat and cold stress activate the hypothalamic–pituitary–adrenal (HPA) axis, stimulating cortisol secretion, a widely used physiological stress indicator [8]. However, cortisol response thresholds vary with species, breed, age, and physiological status [17,22,46]. In the present study, cortisol levels increased at 18.06 °C during cold exposure, whereas elevated plasma cortisol was reported only at −4 °C in beef calves [46] and at 9.5 °C in Sahiwal zebu calves [8]. Under heat stress, the cortisol breakpoint occurred at 28.15 °C (THI 77.36) in the present study, compared with a UCT of 32.2 °C at 60% relative humidity (THI 80.6) in Holstein calves [47], an upper critical THI of 88.8 based on salivary cortisol in Holstein dairy calves [22], and a UCT of 32.29 °C (THI 84.29) in zebu calves [8]. These findings indicate that buffalo calves exhibit a higher LCT and lower UCT than zebu cattle, suggesting that buffaloes initiate endocrine stress responses at comparatively lower cold and heat stress levels. The increase in neutrophil-to-lymphocyte ratio during heat exposure may also reflect cortisol-mediated stress responses [8].

The relatively wider 95% confidence intervals for the estimated breakpoints of ROS, SOD and cortisol indicate greater uncertainty in these estimates; therefore, these thresholds should be interpreted cautiously as approximate transition points rather than precise biological limits.

HSP70 and HSP90 are key molecular indicators of thermal stress [10]. In the present study, HSP70 and HSP90 transcript abundance decreased progressively with decreasing exposure temperature, but neither exhibited a distinct LCT, indicating a gradual rather than threshold-dependent molecular response during cold exposure. Similar observations have been reported previously, where cold-induced HSP responses were less pronounced than physiological or endocrine changes [8,48]. In contrast, clear UCTs for HSP70 and HSP90 occurred under heat stress (THI 84–85), approximately 6 °C higher than the physiological UCT (28.6 °C). The higher breakpoint temperatures of HSP70 and HSP90 compared with the physiological responses indicate that these molecular responses became evident at relatively greater thermal loads. The higher HSP70 and HSP90 breakpoint temperatures than those reported in adult zebu cattle [17] suggest that detectable HSP transcriptional responses occurred at relatively greater thermal loads in the present calves; however, differences in age, breed and experimental conditions should be considered when making such comparisons. The higher HSP70 and HSP90 breakpoints relative to RR, RT and cortisol suggest that these molecular responses were less sensitive to the lower levels of thermal challenge examined in the present study.

**Figure 10:**
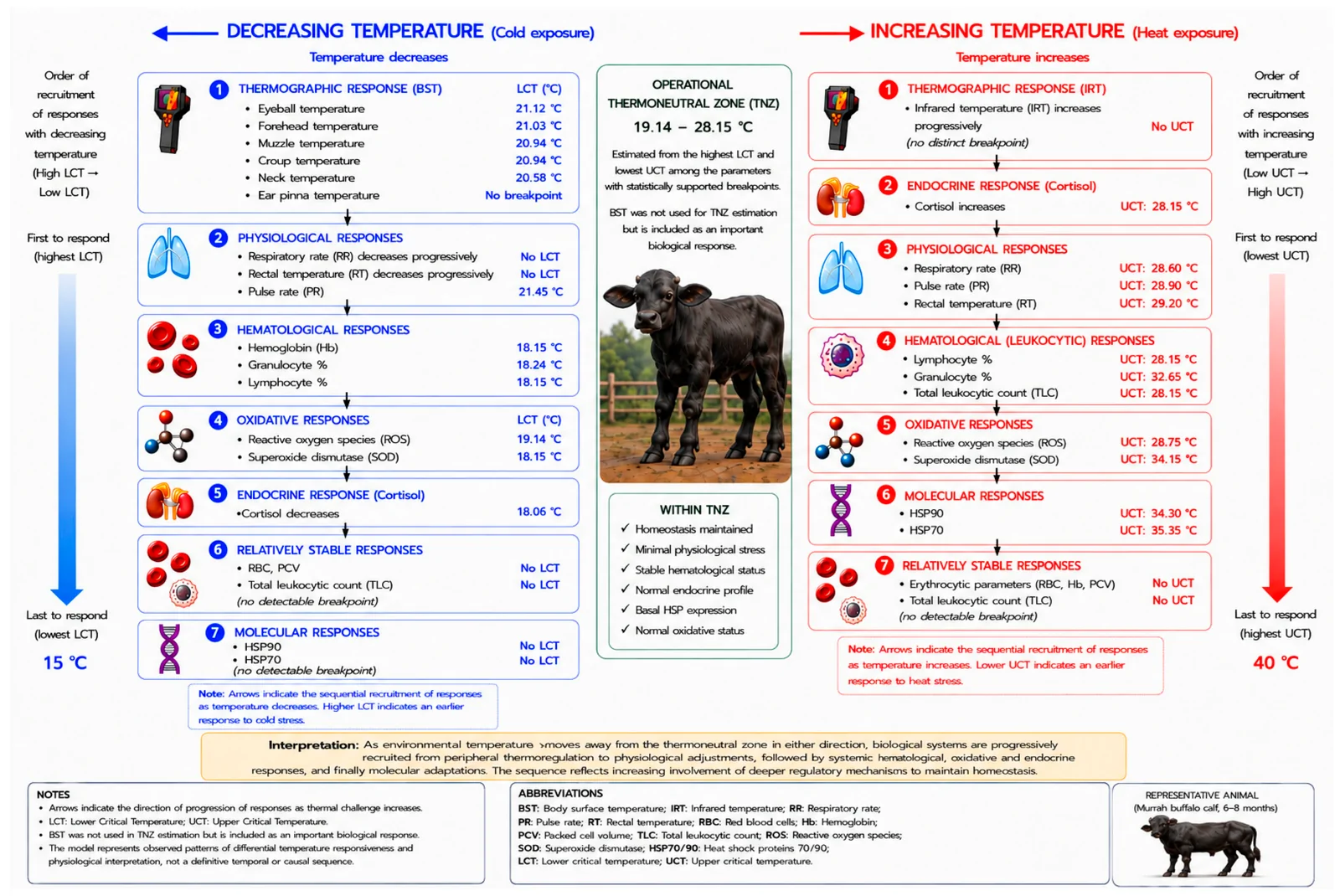
Relative temperature sensitivity and threshold responses of physiological, hematological, endocrine, oxidative, and molecular indicators in Murrah buffalo calves under decreasing and increasing environmental temperatures. The left and right sections represent decreasing- and increasing-temperature exposure, respectively; arrows indicate the direction of temperature change, while the grouped indicators and labelled temperatures represent the corresponding response thresholds identified by segmented regression.

In the present study, Table 3 and Figure 10 summarize the relative sensitivity and temperature thresholds of the assessed biological responses in buffalo calves under increasing and decreasing environmental temperatures. Although the sensitivity and temperature thresholds of individual parameters were discussed earlier, the table highlights that the overall response pattern differs between cold and heat stress. Breakpoint analysis showed that PR, haemoglobin concentration, granulocyte and lymphocyte percentages, BSTs (eyeball, croup, neck, muzzle, and forehead), ROS, SOD, and cortisol showed statistically supported breakpoints during cold stress. In contrast, PR, RR, RT, granulocyte and lymphocyte percentages, ROS, SOD, cortisol, HSP70, and HSP90 responded to heat stress. Red blood cell count and packed cell volume did not show statistically supported breakpoints under either cold or hot conditions. Importantly, the absence of a breakpoint does not necessarily indicate the absence of a temperature-related response. Some parameters changed progressively with increasing or decreasing temperature without a distinct change in slope and therefore did not yield a discrete breakpoint, whereas others remained relatively stable across the tested temperature range. Thus, the difference among parameters in exhibiting breakpoints may reflect differences in their response pattern to thermal challenge, with some showing a distinct threshold and others responding continuously or remaining relatively stable. Lymphocyte percentage and cortisol concentration showed relatively early statistically supported responses to increasing ambient temperature. Under cold stress, BST represented the early peripheral response, reflecting rapid physical thermoregulation through peripheral vasoconstriction, whereas changes in PR and cortisol represented subsequent systemic responses, indicating activation of metabolic and endocrine heat-producing mechanisms once peripheral insulation became insufficient.

It is important to note that the sequential exposure design was intended to characterize both temperature-specific responses and physiological adjustments during progressive changes in environmental temperature. However, acclimation and carry-over effects associated with sequential exposure cannot be completely excluded and may have influenced some responses; therefore, the identified thresholds should be interpreted within the context of this experimental design.

Based on available literature, the present study integrates physiological, hematological, biochemical, oxidative, and molecular indicators to determine the TNZ in Murrah buffalo calves. Determination of TNZ solely on the basis of RT may not adequately represent the true thermoneutral limits, as several physiological responses occur before changes in RT during both cold and heat exposure. BST showed an early and sensitive response to environmental temperature; however, as a peripheral measure, it is strongly influenced by environmental heat exchange and therefore does not directly represent core homeostatic stability. Thus, BST was considered useful for characterizing the thermal response but was not used as a primary determinant of the TNZ boundaries. Brody (1956) described the comfort zone as approximately 1–15 °C for European cattle and 10–27 °C for zebu cattle based on physiological and thermoregulatory indicators including respiratory rate, rectal and skin temperatures, sweating, and heat production [7]. Based on the integration of multiple physiological, hematological, biochemical, endocrine, and molecular indicators, the TNZ of Murrah buffalo calves was estimated to range from 19.14 to 28.15 °C. The resulting range should be interpreted as an operational estimate derived from the integrated responses observed under the present experimental conditions, rather than as a universal biological limit for Murrah buffalo calves. Previous studies have reported that the TNZ of calves may range from 0 to 26 °C, depending on species, breed, age, nutritional status, hair coat, and climatic conditions [49,50,51,52]. The TNZ reported for Sahiwal calves is wider than that observed for Murrah buffalo calves, indicating greater thermal resilience in zebu calves and a higher susceptibility of buffalo calves to both heat and cold stress. Murrah buffalo calves operate within a narrower comfort window because both their heat dissipation and cold insulation capacities are structurally constrained, making them physiologically sensitive to even moderate thermal deviations.

### Limitations

This study was conducted under controlled climatic chamber conditions using a limited number of Murrah buffalo calves, which represents an inherent constraint of intensive physiological experimentation. Although the repeated-measure design allowed each animal to serve as its own control and enabled high-resolution assessment of thermal responses across defined environmental gradients, the relatively small sample size may limit population-level generalization of the findings. Formal diagnostic assessments of residual normality and homoscedasticity were not performed for the SegReg models. Furthermore, because the same calves were repeatedly evaluated across thermal conditions, within-animal dependence was not explicitly modelled in the SegReg analysis, including the ANOVA-based significance component of the procedure. These limitations should be considered when interpreting the statistical significance and uncertainty of the estimated breakpoints. The sequential exposure to heat and cold phases could introduce potential order or acclimatization effects despite provision of recovery periods. Consequently, the TNZ and breakpoint estimates presented here should be regarded as operational physiological reference values rather than absolute biological boundaries. Future studies involving larger, more homogeneous populations and field validation under commercial production systems are warranted to refine and confirm these thresholds and TNZ.

## 5. Conclusions

This study demonstrates that Murrah buffalo calves exhibit distinct physiological, hematological, oxidative, endocrine, and molecular responses under both cold and heat stress conditions. Integration of multiple biological indicators provided a more accurate estimate of the TNZ (19.14–28.15 °C) compared with reliance on RT alone, identifying an upper critical threshold of approximately THI 78. However, the precision of the estimated TNZ is influenced by the uncertainty associated with the individual threshold estimates and should therefore be interpreted cautiously. The reported thresholds represent controlled experimental estimates and should be validated in larger populations before broad generalization. These defined thermal thresholds provide useful reference points for automated climate-control systems and early warning farm management strategies aimed at preventing thermal stress and safeguarding calf welfare and productivity. Overall, the findings highlight the narrow thermoneutral range of buffalo calves, emphasizing the need for proactive environmental management, with cooling interventions initiated earlier during summer and insulation support provided sooner in winter than typically required for cattle.

## Figures and Tables

**Table 1:** Temperature, relative humidity and temperature-humidity index (THI) during the distinct phases in the psychrometric chamber.

Microclimatic Conditions During Day (0600 h to 1800 h)			Microclimatic Conditions During Night (1800 h to 0600 h)			Carbon Dioxide Concentration (ppm)
Temperature (℃)	Relative Humidity (%)	THI	Temperature (℃)	Relative Humidity (%)	THI	
**Phase 1**						
25	65	73.49	25 °C	65	73.49	800 ± 50
28 °C	60	77.20	25 °C	65	73.49	800 ± 50
31 °C	55	80.62	25 °C	65	73.49	800 ± 50
34 °C	50	83.74	25 °C	65	73.49	800 ± 50
37 °C	48	87.21	25 °C	65	73.49	800 ± 50
40 °C	45	90.32	25 °C	65	73.49	800 ± 50
**Phase 2**						
24 °C	70	72.45	24 °C	70	72.45	800 ± 50
21 °C	70	67.96	24 °C	70	72.45	800 ± 50
18 °C	80	63.62	24 °C	70	72.45	800 ± 50
15 °C	85	58.98	24 °C	70	72.45	800 ± 50

**Table 2:** The primer sequences, amplicon sizes and accession numbers of different genes.

Gene	Sequence (5′–3′)	Length	Amplicon Size	Accession No
HSP70	Forward: AACATGAAGAGCGCCGTGGAGG Reverse: GTTACACACCTGCTCCAGCTCC	22	171	XM_006041893
HSP90	Forward: CTGTCATCAGCAGTGGG	17	75	XM_025271500
	Reverse: ACATGCCAACAGGATCTAC	19		
GAPDH	Forward: CATCACTGCCACCCAGAAGACTG Reverse: ATGCCAGTGAGCTTCCCGTTCAG	23	153	XM_006065800.4

**Table 3:** The lower and upper critical THIs for different stress parameters in *Murrah* buffalo calves.

S. No.	Parameter	Lower THI Threshold	SE of BP (Lower THI Threshold)	Upper Threshold THI	SE of BP (Upper Threshold THI)
1	Respiratory Rate (Breaths/min)	No Breakpoint	—	78.03	0.940
2	Pulse Rate (Beats/min)	64.500	1.03	78.37	1.09
3	Rectal Temperature (°C)	No Breakpoint	—	78.71	1.35
4	Red Blood Cells	No Breakpoint	—	No Breakpoint	—
5	Haemoglobin	63.83	1.45	No Breakpoint	—
6	PCV	No Breakpoint	—	No Breakpoint	—
7	Total Leukocytic Count	No Breakpoint	—	77.36	5.52
8	Granulocyte %	63.96	0.651	82.41	39.20
9	Lymphocyte %	63.83	0.313	77.36	0.496
10	Eyeball temperature	68.14	0.488	No Breakpoint	—
11	Ear pinna temperature	No Breakpoint	—	No Breakpoint	—
12	Croup temperature	67.87	1.46	No Breakpoint	—
13	Neck temperature	67.60	2.00	No Breakpoint	—
14	Muzzle temperature	68.00	1.34	No Breakpoint	—
15	Forehead temperature	68.00	0.446	No Breakpoint	—
16	Reactive oxygen species (mg/mL)	65.31	1.09	78.03	19.50
17	Superoxide dismutase (ng/mL)	63.83	23.90	83.76	15.00
18	Cortisol (ng/mL)	63.69	13.30	77.36	5.52
19	*HSP*70	No Breakpoint	—	85.44	1.35
20	*HSP*90	No Breakpoint	—	84.33	2.17

## Data Availability

The original contributions presented in this study are included in the article. Further inquiries can be directed to the corresponding authors.

## Supplementary Materials

The following supporting information can be downloaded at: https://www.mdpi.com/article/10.3390/ani16182895/s1, Table S1: Complete SegReg statistical parameters for temperature–response relationships during decreasing-temperature exposure (24–15 °C). Table S2: Complete SegReg statistical output for response variables during increasing-temperature exposure (25–40 °C).
