# Supplementary material for: Integrated Biological Responses Define the Thermoneutral Zone of Murrah Buffalo Calves in a Subtropical Climate
Source: Animals (Basel). 2026 Sep 14;16(18):2895. doi: 10.3390/ani16182895 (PMC13603437; doi:10.3390/ani16182895)
Supplement: Supplementary file 1 [file animals-16-02895-s001.zip › animals-4508452-supplementary.pdf]

Table S1: Complete SegReg statistical parameters for temperature–response relationships during decreasing-temperature exposure (24–15 °C).

Response variable	Function type	n	Mean X	Mean Y	Breakpoint (°C)	SE_BP (°C)	95% CI of BP (°C)	Slope(s) (A)	Intercept(s) (C)	CE	SD regression coefficient	SD of Y	SD residual Y	SD of X
**RR**	1	24	19.5	10.4	—	—	—	0.25	5.5	0.387	0.067	1.38	1.08	3.43
**PR**	4	24	19.5	34.8	18.6	0.634	—	1.51	8.37	0.562	0.342	3.06	2.45	3.43
**RT**	1	24	19.5	37.1	—	—	—	0.181	33.6	0.569	0.034	0.822	0.54	3.43
**Eyeball temperature**	4	24	19.5	30.9	21.12	0.297	—	0.4	23.5	0.326	0.14	1.63	1.39	3.43
**Muzzle temperature**	4	24	19.5	20.8	20.94	0.79	—	2.77	-31.1	0.952	0.321	6.79	2.97	3.43
**Ear pinna temperature**	1	24	19.5	17.5	—	—	—	1.62	-14	0.943	0.085	5.71	1.36	3.43
**Neck temperature**	4	24	19.5	29.8	20.58	1.29	—	0.919	12.7	0.726	0.212	2.53	1.68	3.43
**Croup temperature**	4	24	19.5	26.7	20.94	0.99	—	1.33	1.82	0.892	0.2	3.5	1.86	3.43
**Forehead temperature**	4	24	19.5	25.7	21.03	0.236	—	1.3	1.3	0.885	0.126	3.49	1.62	3.43
**PCV**	0	24	19.5	29.8	—	—	—	0	29.8	0.033	0.208	3.41	3.35	3.43
**HGB**	4	24	19.5	9.01	18.15	0.945	—	0.933	-7.16	0.667	0.219	1.79	1.46	3.43
**RBC**	0	24	19.5	6.18	—	—	—	0	6.18	0.018	0.036	0.579	0.573	3.43
**TLC**	1	24	19.5	9.17	—	—	—	0.641	-3.33	0.582	0.116	2.88	1.86	3.43
**LY**	4	24	19.5	5.39	18.15	0.189	—	0.787	-8.24	0.534	0.277	1.69	1.36	3.43
**GR**	4	24	19.5	3.59	18.24	0.404	—	0.675	-8.14	0.573	0.141	1.26	0.876	3.43
**ROS**	4	24	19.5	263	19.14	1.09	—	-77.7	1650	0.724	20.8	158	102	3.43
**SOD**	8	24	19.5	8.28	18.15	23.9	—	-1.02	25.2	0.565	0.329	2.59	2.41	3.43
**Cortisol**	6	24	19.5	52.5	18.06	4.2	—	0	43.6	0.894	0.484	11.3	9.15	3.43
**HSP70**	1	24	19.5	113	—	—	—	-27.4	648	0.946	1.4	96.7	22.6	3.43
**HSP90**	1	24	19.5	13.8	—	—	—	-2.74	67.3	0.875	0.221	10	3.55	3.43

Abbreviations: RR, respiratory rate; PR, pulse rate; RT, rectal temperature; PCV, packed cell volume; HGB, haemoglobin; RBC, red blood cell count; TLC, total leukocyte count; LY, lymphocytes; GR, granulocytes; ROS, reactive oxygen species; SOD, superoxide dismutase; HSP70, heat shock protein 70; HSP90, heat shock protein 90; BP, breakpoint; SE, standard error; CI, confidence interval; CE, coefficient of explanation; SD, standard deviation; n, number of observations; X, independent variable (temperature); Y, dependent/response variable; A, slope; C, intercept; —, not available/not applicable.

Table S2: Complete SegReg statistical output for response variables during increasing-temperature exposure (25–40 °C).

Response variable	Function type	n	Mean X	Mean Y	Breakpoint (°C)	SE_BP (°C)	95% CI of BP (°C)	Slope (A)	Intercept (C)	CE	SD regression coefficient	SD of Y	SD residual Y	SD of X
**RR**	3	36	32.5	39.4	28.6	0.843	—	4.51	-107	0.862	0.31	25.3	9.38	5.2
**PR**	3	36	32.5	50.1	28.9	0.892	—	2.44	-29.3	0.809	0.203	14.1	6.16	5.2
**RT**	3	36	32.5	38.1	29.2	1.16	—	0.133	33.8	0.739	0.0135	0.801	0.409	5.2
**Eyeball temperature**	1	36	32.5	35.7	—	—	—	0.473	20.3	0.859	0.033	2.66	0.999	5.2
**Ear pinna temperature**	1	36	32.5	32.5	—	—	—	1.05	-1.53	0.907	0.058	5.72	1.74	5.2
**Muzzle temperature**	1	36	32.5	32.5	—	—	—	0.878	3.94	0.911	0.047	4.78	1.43	5.2
**Croup temperature**	1	36	32.5	34.7	—	—	—	0.719	11.4	0.905	0.04	3.93	1.21	5.2
**Forehead temperature**	1	36	32.5	34.3	—	—	—	0.717	10.9	0.886	0.044	3.96	1.34	5.2
**Neck temperature**	1	36	32.5	35.7	—	—	—	0.555	17.6	0.836	0.042	3.16	1.28	5.2
**Cortisol**	5	36	32.5	45	28.15	5.52	16.94–39.36	0	32.0 / 51.5	0.151	0.56	18.4	17	5.2
**ROS**	6	36	32.5	272	28.75	4.67	25.19–44.15	0.646	251	0.00065	4.34	131	131	5.2
**SOD**	6	36	32.5	9.16	34.15	6.55	20.85–47.45	-3.27	138	0.619	0.147	4.58	4.45	5.2
**RBC**	0	36	32.5	5.85	—	—	—	-0.0515	7.53	0.051	0.038	1.19	1.16	5.2
**PCV**	0	36	32.5	27.9	—	—	—	-0.219	35	0.043	0.177	5.48	5.36	5.2
**HGB**	0	36	32.5	9.46	—	—	—	-0.103	12.8	0.071	0.064	2.01	1.94	5.2
**TLC**	5	36	32.5	11.9	28.15	0.753	—	0	10.8 / 12.4	0.153	0.063	2	1.92	5.2
**LY**	5	36	32.5	7.43	28.15	0.496	—	0.124	3.38	0.13	0.055	1.79	1.67	5.2
**GR**	6	36	32.5	4.08	32.65	14.2	—	-0.019	4.7	0.0099	0.032	0.983	0.978	5.2
**HSP70**	3	36	32.5	58.2	35.35	1.32	—	11	-299	0.642	1.41	71.3	42.7	5.2
**HSP90**	3	36	32.5	16.1	34.3	2.11	—	3.69	-104	0.556	0.565	25.7	17.1	5.2

Abbreviations: RR, respiratory rate; PR, pulse rate; RT, rectal temperature; PCV, packed cell volume; HGB, haemoglobin; RBC, red blood cell count; TLC, total leukocyte count; LY, lymphocytes; GR, granulocytes; ROS, reactive oxygen species; SOD, superoxide dismutase; HSP70, heat shock protein 70;

HSP90, heat shock protein 90; BP, breakpoint; SE, standard error; CI, confidence interval; CE, coefficient of explanation; SD, standard deviation; n, number of observations; X, independent variable (temperature); Y, dependent/response variable; A, slope; C, intercept; —, not available/not applicable.
